# Clinical Significance of Ultrasound Elastography and Fibrotic Focus and Their Association in Breast Cancer

**DOI:** 10.3390/jcm11247435

**Published:** 2022-12-15

**Authors:** Na-Rang Lee, Hoon-Kyu Oh, Young-Ju Jeong

**Affiliations:** 1 Department of Surgery, School of Medicine, Daegu Catholic University, Daegu 42472, Republic of Korea; 2 Department of Pathology, School of Medicine, Daegu Catholic University, Daegu 42472, Republic of Korea

**Keywords:** elastography, breast imaging, fibrotic focus, breast cancer, tissue stiffness, prognostic biomarker

## Abstract

(1) Background: Ultrasound (US) elastography is an imaging technology that reveals tissue stiffness. This study aimed to investigate whether fibrotic focus (FF) affects elastographic findings in breast cancer, and to evaluate the clinical significance of US elastography and FF in breast cancer. (2) Methods: In this study, 151 patients with breast cancer who underwent surgery were included. Strain elastography was performed and an elasticity scoring system was used to assess the findings. The elasticity scores were classified as negative, equivocal, or positive. FF was evaluated in the surgical specimens. Medical records were reviewed for all patients. (3) Results: Elastographic findings were equivocal in 30 patients (19.9%) and positive in 121 patients (80.1%). FF was present in 68 patients (46.9%). There was no correlation between elastographic findings and FF. Older age, larger tumor size, lymph node metastasis, and higher tumor stage were associated with positive elastographic results. FF showed a positive correlation with age, postmenopausal status, tumor size, lymphovascular invasion, lymph node metastasis, tumor stage, and intratumoral and peritumoral inflammation. (4) Conclusions: Our study showed that positive elastographic results and FF were associated with poor prognostic factors for breast cancer. FF did not affect the elastographic findings of this study.

## 1. Introduction

Ultrasonography (US) is a medical imaging technique widely used in clinical practice [1]. Breast US shows detailed images of the inside of the breast using high-frequency sound waves. In addition to breast US, US elastography provides additional information for the assessment and characterization of breast masses by imaging tissue stiffness [1,2]. Previous studies have shown that fat, normal glandular tissue, fibrous tissue, and tumor tissue exhibit different elastic moduli at different strain levels [3]. Malignant lesions tend to be harder than benign lesions, and in this regard, malignant breast tumors can be differentiated by US elastography [4].

Recently, US elastographic features have been added to the fifth edition of the Breast Imaging Reporting and Data System (BI-RADS) US lexicon [5]. The incorporation of positive or negative US elastographic results into B-mode US findings influences the BI-RADS classification of breast lesions and increases the specificity and diagnostic accuracy of breast lesions [5,6].

Malignant breast tumors show increased stiffness on US elastography compared to benign lesions, but several studies have shown different elastographic findings in invasive breast cancer [7,8,9]. Several factors have been suggested in previous studies regarding the cause of the difference in elasticity in breast cancer, but this remains unclear [7,8,9,10,11]. In a previous study, myofibroblasts were suggested to be one of the factors affecting the elasticity of breast cancer [11]. The authors suggested that myofibroblasts produce collagen and extracellular matrix (ECM) proteins and constitute a desmoplastic reaction, which may affect the stiffness or elasticity of breast cancer [11]. The authors showed a positive correlation between US elastography scoring and myofibroblasts in breast cancer [11].

Aberrant ECM remodeling can lead to pathological fibrosis and an increased risk of cancer [12]. In the tumor micro-environment, ECM remodeling induces ECM stiffness [12], and contributes to fibrotic changes [13]. Fibrotic focus (FF) is defined as a mixture of fibroblasts and various amounts of collagen fibers [14]. In this regard, FF may affect ECM stiffness in breast cancer and thus may influence the results of US elastography.

Our previous study and several other studies have demonstrated that FF is associated with poor prognostic markers for breast cancer [14,15,16,17]. Some studies have reported an association between US elastography results and clinicopathological factors in breast cancer [7,8,9,10]. However, there have been no studies on the relationship between FF and US elastography in breast cancer. This study aimed to investigate whether FF affects the results of US elastography in breast cancer and to evaluate the clinical significance of US elastography and FF in breast cancer. We analyzed the association between elastographic findings, FF, and clinicopathological factors.

## 2. Materials and Methods

This retrospective study included patients who underwent surgical treatment for breast cancer at Daegu Catholic University Hospital in Daegu, Republic of Korea between 2013 and 2017. Patients who received neoadjuvant chemotherapy prior to surgery or underwent palliative surgery for breast cancer were excluded. Ethical approval for this study was obtained from the Institutional Review Board of Daegu Catholic University Hospital (CR-22-026). Our Institutional Review Board waived the requirement for written informed consent for the study, according to the deliberation criteria. Medical records were reviewed for all patients. Clinicopathological features included age, menopausal status, surgical methods, tumor size, histologic grade, lymphovascular invasion, regional lymph node metastasis status, estrogen receptor status, progesterone receptor (PR) status, human epidermal growth factor receptor 2 (HER2), and Ki-67 labeling index. The tumor stage was assessed according to the eighth edition of the American Joint Committee on Cancer Staging Manual for Breast Cancer.

Mammography, breast US with strain elastography (SE), breast magnetic resonance imaging, and positron emission tomography-computed tomography were performed pre-operatively in all patients. US elastographic images and conventional B-mode images, including the tumor and surrounding tissue, were obtained using a Philips iU22 apparatus (Philips Ultrasound; Philips Healthcare, Bothell, WA, USA) with a linear array transducer (L12-5, 12–5 MHz) by a single radiologist with 15 years of experience in breast US.

The five-point scale elasticity score proposed by Itoh et al. [18] was used to assess elastographic findings. A score of 1 indicated a lesion evenly shaded in green for the entire hypoechoic lesion; a score of 2 indicated a hypoechoic lesion with a mosaic pattern of green and blue; a score of 3 indicated a hypoechoic lesion with blue at the central part and green at the periphery; a score of 4 indicated an entire hypoechoic lesion of blue, but its surrounding area was not included; and a score of 5 indicated an entire hypoechoic lesion of blue, including its surrounding area [18]. We classified the elasticity scores into three categories: a score of 1 as negative (soft lesion), scores of 2 and 3 as equivocal (intermediate lesion), and scores of 4 and 5 as positive (hard lesion).

**Figure 1:**
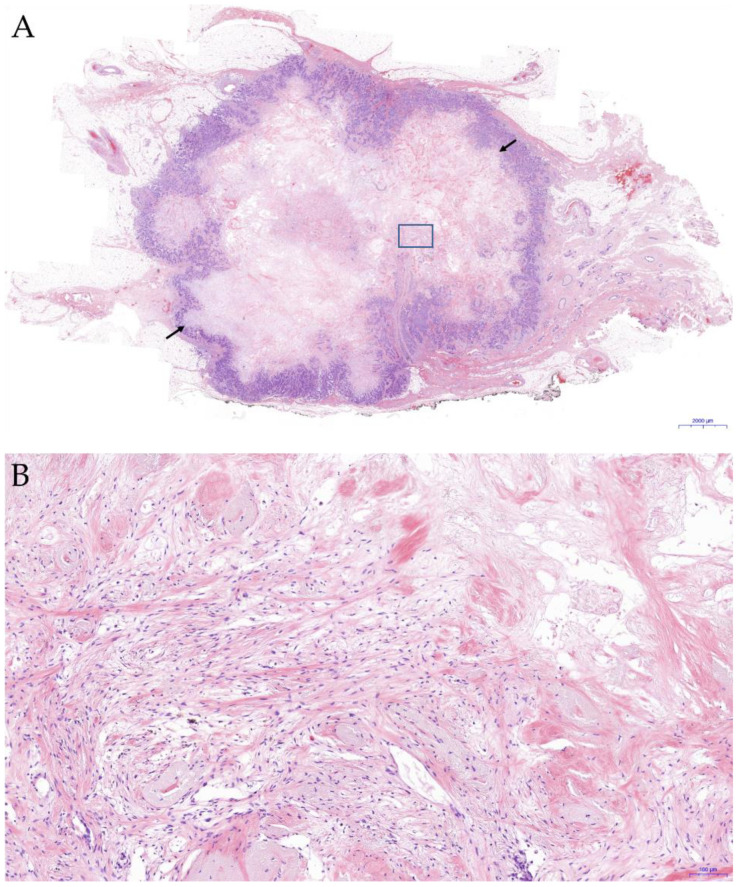
Representative histology of fibrotic focus (FF) in breast cancer. (**A**) Arrows indicate area of FF in invasive breast cancer tissues (H&E; magnification, ×6). The rectangle indicate the magnified area of FF shown in (**B**). (**B**) A mixture of fibroblast-like spindle cells and variable collagen fibers at the center of FF. (H&E; magnification, ×100).

Formalin-fixed and paraffin-embedded (FFPE) specimens of primary breast cancer were stained with hematoxylin and eosin, and examined microscopically by an experienced pathologist. FF was diagnosed when there was a scar-like area, or radially expanding fibrous bands consisting of fibroblasts and collagen fibers within the tumor surrounded by a highly cellular zone of infiltrating carcinoma cells [16]. The size and grade of FF within the tumor were assessed in FFPE specimens. Positive FF was defined as fibrous lesions ≥ 1 mm that are characteristic of FF, such as fibroblasts arranged in irregular or storiform patterns showing increased fibroblast cellularity and/or collagenization (Figure 1) [16].

Lymphocyte infiltration was evaluated within the tumor boundary (intratumoral) and at the edge of the tumor boundary (peritumoral) as previously described [15]. Intratumoral and peritumoral inflammation was evaluated semi-quantitatively as 0 for no or scant lymphocytes, 1 for a few scattered lymphocyte infiltrations, 2 for scattered lymphocyte aggregation, and 3 for diffuse and dense lymphocytes. Scores 1, 2, and 3 were designated as positive and 0 as negative [15].

Statistical analyses were performed using SPSS Statistics (version 25.0; IBM Corp., Armonk, NY, USA). The association between the elastographic findings and FF was analyzed using a two-sided Pearson’s chi-squared test. To compare categorical variables, the chi-squared test or Fisher’s exact test was used. Student’s *t*-test or the nonparametric Mann–Whitney U-test was used to analyze continuous data, including age, mean tumor size, FF size, and maxSUV on PET-CT. A multivariate logistic regression analysis model was used to analyze the independent predictors for FF and US elastography findings, and the odds ratios and 95% confidence intervals were calculated. The *p*-value threshold of the variables included in the multivariate analysis was *p*-value < 0.2 in the univariate analysis. The Kaplan–Meier method with the log-rank test was used to compare recurrence-free survival (RFS) and overall survival (OS) according to the elastographic findings and FF. For all analysis results, a *p*-value of < 0.05 was considered statistically significant, and all *p*-values were two-sided.

## 3. Results

### 3.1. Clinicopathological Characteristics of the Patients

A total of 151 patients were included in this study. All the patients were diagnosed with invasive breast cancer. The most common histological type was invasive ductal carcinoma not otherwise specified in 134 patients (88.7%), followed by lobular carcinoma in seven patients, papillary carcinoma in four patients, metaplastic carcinoma in three patients, mucinous carcinoma in two patients, and micropapillary carcinoma in one patient. The mean age of the patients was 57.6 ± 11.4 years (range, 25–82 years). The elastographic findings were equivocal in 30 patients (19.9%) and positive in 121 patients (80.1%). No soft lesions were observed on US elastography in patients with breast cancer in this study. FF was present in 68 patients (46.9%). Table 1 shows the clinicopathological characteristics of the patients. The median follow-up period was 67 months (range 8–101 months). During the follow-up period, tumor recurrence occurred in 15 patients (9.9%), and breast cancer-related deaths occurred in 10 patients (6.6%).

### 3.2. Association between US Elastography and FF

There was no correlation between the elastographic findings using the elasticity score and FF (*p* = 0.633) (Table 2). The size and grade of the FF also did not show any association with the elastographic findings (*p* = 0.363 and *p* = 0.439, respectively).

### 3.3. Association between US Elastography and Clinicopathological Features

Compared with intermediate lesions by US elastography, hard lesions (positive results) were significantly correlated with older age, larger tumor size, lymph node metastasis, and higher tumor stage (*p* = 0.040, *p* = 0.004, *p* = 0.043, and *p* = 0.042, respectively) (Table 2). In the multivariate analysis, positive elastographic results were significantly associated with larger tumor size (*p* = 0.003) (Table 3). There was no association between US elastography findings, microcalcification, or other immunohistochemical findings of breast cancer.

**Figure 2:**
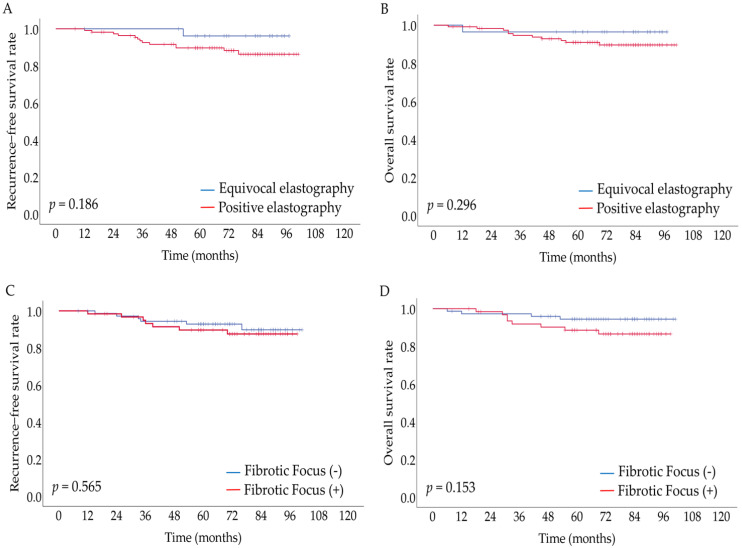
Association between ultrasound (US) elastography and fibrotic focus (FF) and patient outcomes in breast cancer. (**A**) Recurrence−free survival (RFS) according to US elastography, (**B**) overall survival (OS) according to US elastography, (**C**) RFS according to FF, (**D**) OS according to FF. Kaplan−Meier survival analysis with the log-rank test was used, and a *p*−value of <0.05 was considered statistically significant.

The five-year RFS and OS tended to be longer in the equivocal elastographic results than in the positive elastographic results, but the differences were not statistically significant (96.2% vs. 89.7%, *p* = 0.186; 96.4% vs. 90.9%, *p* = 0.296, respectively) (Figure 2A,B).

### 3.4. Correlation between FF and Clinicopathological Features

FF was positively correlated with age (*p* = 0.002), postmenopausal status (*p* = 0.008), tumor size (*p* < 0.001), lymphovascular invasion (*p* = 0.015), lymph node metastasis (*p* = 0.029), tumor stage (*p* = 0.004), and intratumoral and peritumoral inflammation (*p* < 0.001 and *p* = 0.003, respectively) (Table 4). In the multivariate analysis, positive FF was independently associated with older age, larger tumor size, and positive intratumoral inflammation (*p* = 0.034, *p* = 0.001, and *p* = 0.007, respectively) (Table 5). There was no significant association between FF and patient outcomes (Figure 2C,D).

## 4. Discussion

Fibrosis is the formation of excess connective tissue that causes stromal thickening and scarring [19,20]. Tumor fibrosis has been recognized to be associated with uncontrolled inflammation [19,21], and characterized by chronic inflammation and aberrant ECM remodeling [22]. This altered tumor micro-environment could affect tissue dynamics as measured by US elastography, indicating tissue stiffness [23]. We hypothesized that FF is associated with tissue stiffness and affects the US elastography results in breast cancer. In this study, we assessed the elastographic findings using a five-point scale elasticity score and found no correlation between the elastographic findings and FF. This is a novel study investigating the association between FF and US elastography in breast cancer.

Previous studies have suggested several factors related to US elastography in breast cancer [7,8,9,10,11,24], but few studies have focused on fibrosis. Hao et al. suggested that myofibroblasts affect tissue stiffness and are associated with US elastography in breast cancer [11]. In fibrotic tumor stroma, the increased concentration of collagen and altered alignment of collagenous fibers can lead to an increase in tissue stiffness [22,24]. However, Liu et al. demonstrated the heterogeneity of mechanical properties using US elastography in breast cancer and suggested that the distribution of stiffness within and around the tumor tissue is heterogeneous [24]. It is well known that breast cancer comprises several components that include cancerous epithelial cells, cells of different lineages, tumor microvasculature, and the ECM [24]. These components have different mechanical properties, which may lead to heterogeneity in elastography within the tumor [24]. In our study, the relationship between FF and US elastography may have been underestimated because US elastography of the entire cancer tissue was obtained without measuring the elastography for FF and the other components separately.

US elastography estimates tissue stiffness by monitoring the response of the tissue to mechanical stimuli and measuring the mechanical properties of tissues [23]. According to the nature of the external mechanical stimulus, US elastography techniques are divided into two methods: strain-based (SE or quasi-static methods) and shear-wave-based (shear-wave elastography [SWE] or dynamic methods) [1,2,23]. In SE, tissue stiffness is measured by applying external tissue pressure, such as probe compression or intrinsic mechanical force, whereas shear-wave elastography (SWE) uses an acoustic radiation impulse force created by a focused ultrasound beam to display images of the shear wave speed. In a study comparing SWE and SE in breast lesions, the diagnostic performances of both methods for differentiating benign and malignant breast lesions were similar [25]. However, both methods have advantages and disadvantages, and their sensitivity and specificity differ according to the histological profile of the lesion, tumor grade, and breast thickness [25]. Because only the SE method was used in our study, the expected results may not have been obtained owing to the differences in the techniques. To confirm our results, it is necessary to confirm whether similar results are obtained when both SE and SWE are applied to the same breast cancer.

As the diagnostic usefulness of US elastography in breast cancer has been recognized, recent studies have investigated its relevance to the clinicopathological characteristics of breast cancer. Hayashi et al. used SE to evaluate tumor stiffness and reported that tumor stiffness was significantly correlated with lymph node involvement and invasive tumor size in breast cancer [9]. Previous studies using SWE showed that breast cancers with higher mean elasticity values were significantly associated with poor prognostic features such as high histologic grade, large tumor size, lymph node involvement, histological subtype, and lymphovascular invasion [8,26,27]. Consistent with the results of previous studies, our study also showed a significant correlation between tumor stiffness and poor prognostic features of breast cancer, including older age, larger tumor size, lymph node metastasis, and higher tumor stage. Several studies have demonstrated that the presence of calcifications in breast lesions is correlated with increased mean elasticity values on SWE [28,29,30]. However, the impact of microcalcification on elastography in breast cancer remains unclear. In our study, there was no association between the US elastography findings and microcalcification.

It has been demonstrated that the presence of FF is associated with poor prognostic factors in breast cancer. We also reported the prognostic significance of FF in breast cancer in our previous study [15]. FF was consistently found to be associated with poor prognostic factors, including older age, larger tumor size, lymphovascular invasion, lymph node metastasis, and intratumoral and peritumoral inflammation.

This study had several limitations. First, although several different commercially available US elastography systems are available, only one SE technique was used in this study. The SE method has a potential shortcoming: US elastographic findings may vary depending on US probe compression and may be less reproducible. Furthermore, several SE features such as strain ratio, elasticity score (Tsukuba score), and elastography-to-B-mode size ratio have been proposed in SE, but only the elasticity score was included in the analysis of this study. Additional studies, including other US elastography techniques and various other features, are needed to further clarify the relationship between US elastography and FF. Secondly, because this was a retrospective study, various imaging findings could not be included. Conventional B-mode US findings were not included in the analysis. In our clinical practice, we usually add the elastographic findings to existing B-mode US images to classify breast lesions according to BI-RADS categories. It is acknowledged that the US elastography technique can improve the overall diagnostic performance in the differentiation of benign and malignant lesions when combined with B-mode US [5,6,25]. Although only breast cancer patients were included in this study, it is expected that additional detailed results can be derived by combining elastographic findings with B-mode US findings. Thirdly, the association between histological characteristics and US elastographic findings was not analyzed. Because of the heterogeneity of the mechanical properties of breast cancer [24], histological findings other than FF may affect tissue stiffness. Further studies are needed to elucidate the histological characteristics that influence US elastography findings in breast cancer.

## 5. Conclusions

Our study showed that positive elastographic results indicating tumor stiffness and FF were associated with poor prognostic factors for breast cancer. There was no correlation between the elastographic findings and FF in this study. Further studies on the fibrotic tumor micro-environment and elastography are required to elucidate the clinical significance and relevance of FF and US elastography in breast cancer.

## Figures and Tables

**Table 1:** The clinicopathological characteristics of patients.

Variables	Value (*n* = 151)
Age (years)	
Mean age ± SD, (range)	57.6 ± 11.4 years (25–82)
Menopausal status, *n* (%)	
Premenopausal	48 (31.8)
Postmenopausal	103 (68.2)
Breast surgery method, *n* (%)	
Mastectomy	45 (63.4)
Breast-conserving surgery	26 (36.6)
Tumor size	
Mean size ± SD (range) (cm)	1.6 ± 1.0 (0.1–6.3)
<2 cm, *n* (%)	107 (70.9)
≥2 cm, *n* (%)	44 (29.1)
Histologic grade, *n* (%)	
I	32 (21.2)
II	53 (35.1)
III	66 (43.7)
Lymphovascular invasion, *n* (%)	32 (21.2)
LN metastasis, *n* (%)	36 (24.2)
Extranodal extension, *n* (%)	19 (12.8)
Microcalcification, *n* (%)	70 (46.4)
Stage, *n* (%)	
I	92 (60.9)
II	44 (29.2)
III	15 (9.9)
ER, *n* (%)	
Negative	41 (27.2)
Positive	110 (72.8)
PR, *n* (%)	
Negative	47 (31.1)
Positive	104 (68.9)
HER2 overexpression, *n* (%)	
Negative	127 (84.1)
Positive	24 (15.9)
Molecular subtype, *n* (%)	
Luminal A	53 (35.1)
Luminal B	61 (40.4)
HER2	17 (11.3)
Basal-like	20 (13.2)
Bcl2, *n* (%)	
Negative	35 (23.2)
Positive	116 (76.8)
P53, *n* (%)	
Negative	35 (23.2)
Positive	116 (76.8)
Ki-67, *n* (%)	
<14%	60 (39.7)
≥14%	91 (60.3)
EGFR, *n* (%)	
Negative	119 (78.8)
Positive	32 (21.2)
Positive fibrotic focus, *n* (%)	68 (46.9)
Positive intratumoral inflammation, *n* (%)	110 (75.3)
Positive peritumoral inflammation, *n* (%)	117 (80.1)
Elastography, *n* (%)	
Equivocal	30 (19.9)
Positive	121 (80.1)
Adjuvant chemotherapy	88 (58.7)
Adjuvant endocrine therapy	115 (76.2)
Recurrence, *n* (%)	15 (9.9)
Breast cancer-related death, *n* (%)	10 (6.6)

SD = standard deviation; LN = lymph node; ER = estrogen receptor; PR = progesterone receptor; HER2 = human epidermal growth factor receptor 2; EGFR = epidermal growth factor receptor.

**Table 2:** Association between the elastographic findings and fibrotic focus, and clinicopathological characteristics of breast cancer.

Variables	Elastography		*p*-Value
	Equivocal	Positive	
Fibrotic focus, *n* (%)			0.633
Negative	16 (57.1)	61 (52.1)	
Positive	12 (42.9)	56 (47.9)	
Fibrotic focus grade, *n* (%)			0.439
0	16 (57.1)	61 (53.5)	
1	2 (7.1)	8 (7.0)	
2	9 (32.1)	28 (24.6)	
3	1 (3.6)	17 (14.9)	
Fibrotic focus size (cm), mean ± SD	0.2 ± 0.4	0.3 ± 0.5	0.363
Intratumoral inflammation, *n* (%)			0.963
Negative	7 (25.0)	29 (24.6)	
Positive	21 (75.0)	89 (75.4)	
Peritumoral inflammation, *n* (%)			0.767
Negative	5 (17.9)	24 (20.3)	
Positive	23 (82.1)	94 (79.7)	
Age (years), mean ± SD	53.8 ± 12.2	58.6 ± 11.1	0.040 *
Menopausal status, *n* (%)			0.129
Premenopausal	13 (43.3)	35 (28.9)	
Postmenopausal	17 (56.7)	86 (71.1)	
Tumor size (cm), mean ± SD	1.2 ± 08	1.7 ± 1.0	0.004 *
<2 cm, *n* (%)	26 (86.7)	81 (66.9)	0.033 *
≥2 cm, *n* (%)	4 (13.3)	40 (33.1)	
Histologic grade, *n* (%)			0.555
I	5 (16.7)	27 (22.3)	
II	11 36.7)	42 (34.7)	
III	14 (46.7)	52 (43.0)	
Lymphovascular invasion, *n* (%)	5 (16.7)	27 (22.3)	0.498
LN metastasis, *n* (%)	3 (10.0)	33 (27.7)	0.043 *
Microcalcification, *n* (%)	18 (60.0)	52 (43.0)	0.094
Stage, *n* (%)			0.042 *
I	24 (80.0)	68 (56.2)	
II	4 (13.3)	40 (33.1)	
III	2 (6.7)	13 (10.7)	
Molecular subtype, *n* (%)			0.513
Luminal A	12 (40.0)	41 (33.9)	
Luminal B	7 (23.3)	54 (44.6)	
HER2	6 (20.0)	11 (9.1)	
Basal-like	5 (16.7)	15 (12.4)	
MaxSUV on PET-CT, mean ± SD	2.8 ± 3.2	2.8 ± 3.4	0.954

SD = standard deviation; LN = lymph node; HER2 = human epidermal growth factor receptor 2; maxSUV = maximal standardized uptake value; PET-CT = positron emission tomography-computed tomography. * Indicates statistically significant (*p* < 0.05).

**Table 3:** Univariate and multivariate analysis of association between positive elastographic finding and clinicopathological characteristics.

Variables	Univariate Analysis		Multivariate Analysis	
	OR (95% CI)	*p*-Value	OR (95% CI)	*p*-Value
Older age	1.039 (1.001–1.079)	0.044 *	1.021 (0.964–1.082)	0.477
Postmenopause	1.879 (0.826–4.275)	0.133	1.327 (0.363–4.848)	0.669
Large tumor size	2.353 (1.304–4.243)	0.004 *	2.493 (1.355–4.587)	0.003 *
Positive lymph node metastasis	3.453 (0.981–12.157)	0.054	9.621 (0.885–104.604)	0.063
Stage II vs. Stage I, III	3.529 (1.142–10.907)	0.028 *	0.429 (0.071–2.587)	0.429
Stage III vs. Stage I, II	2.294 (0.482–10.914)	0.297	0.075 (0.003–1.757)	0.107
Presence of microcalcifications	0.502 (0.223–1.134)	0.098	0.482 (0.206–1.124)	0.091

OR = odds ratio; CI = confidence interval. * Indicates statistically significant (*p* < 0.05).

**Table 4:** Association between fibrotic focus and clinicopathological characteristics of breast cancer.

Variables	Fibrotic Focus		*p*-Value
	Positive	Negative	
Age (years), mean ± SD	60.6 ± 11.0	54.8 ± 11.2	0.002 *
Menopausal status, *n* (%)			0.008 *
Premenopausal	15 (22.1)	33 (42.9)	
Postmenopausal	53 (77.9)	44 (57.1)	
Tumor size (cm), mean ± SD	2.0 ± 1.0	1.3 ± 1.0	<0.001 *
<2 cm, *n* (%)	38 (55.9)	65 (84.4)	<0.001 *
≥2 cm, *n* (%)	30 (44.1)	12 (15.6)	
Histologic grade, *n* (%)			0.332
I	11 (16.2)	20 (26.0)	
II	26 (38.2)	24 (31.2)	
III	31 (45.6)	33 (42.9)	
Lymphovascular invasion, *n* (%)	20(29.4)	10 (13.0)	0.015 *
LN metastasis, *n* (%)	22 (32.8)	13 (17.1)	0.029 *
Extranodal extension, *n* (%)	11 (16.4)	7 (9.2)	0.195
Microcalcification, *n* (%)	34 (50.0)	36 (46.8)	0.696
Stage, *n* (%)			0.004 *
I	32 (47.1)	57 (74.0)	
II	27 (39.1)	14 (18.2)	
III	9 (13.2)	6 (7.8)	
ER, *n* (%)			0.628
Negative	17 (25.0)	22 (28.6)	
Positive	51 (75.0)	55 (71.4)	
PR, *n* (%)			0.554
Negative	19 (27.9)	25 (32.5)	
Positive	49 (72.1)	52 (67.5)	
HER2 overexpression, *n* (%)			0.909
Negative	57 (83.8)	64 (83.1)	
Positive	11 (16.2)	13 (16.9)	
Molecular subtype, *n* (%)			0.718
Luminal A	21 (30.9)	28 (36.4)	
Luminal B	32 (47.1)	29 (37.7)	
HER2	7 (10.3)	10 (13.0)	
Basal-like	8 (11.8)	10 (13.0)	
Bcl2, *n* (%)			0.545
Negative	17 (25.0)	16 (20.8)	
Positive	51 (75.0)	61 (79.2)	
P53, *n* (%)			0.111
Negative	20 (29.4)	14 (18.2)	
Positive	48 (70.6)	63 (81.8)	
Ki-67, *n* (%)			0.338
<14%	23 (33.8)	32 (41.6)	
≥14%	45 (66.2)	45 (58.4)	
EGFR, *n* (%)			0.827
Negative	54 (79.4)	60 (77.9)	
Positive	14 (20.6)	17 (22.1)	
Positive intratumoral inflammation, *n* (%)	60 (88.2)	50 (64.9)	<0.001 *
Positive peritumoral inflammation, *n* (%)	62 (91.2)	55 (71.4)	0.003 *
MaxSUV on PET-CT, mean ± SD	2.6 ± 2.9	2.6 ± 3.0	0.983
Adjuvant chemotherapy	44 (64.7)	41 (53.9)	0.190
Adjuvant endocrine therapy	54 (79.4)	57 (74.0)	0.445
Recurrence, *n* (%)			0.419
Yes	8 (11.8)	6 (7.8)	
No	60 (88.2)	71 9 (92.2)	
Breast cancer-related death, *n* (%)			0.190
Yes	7 (10.4)	3 (3.9)	
No	60 (89.6)	73 (96.1)	

SD = standard deviation; LN = lymph node; ER = estrogen receptor; PR = progesterone receptor; HER2 = human epidermal growth factor receptor 2; EGFR = epidermal growth factor receptor; maxSUV = maximal standardized uptake value; PET-CT = positron emission tomography-computed tomography; * Indicates statistically significant (*p* < 0.05).

**Table 5:** Univariate and multivariate analysis of association between fibrotic focus and clinicopathological characteristics.

Variables	Univariate Analysis		Multivariate Analysis	
	OR (95% CI)	*p*-Value	OR (95% CI)	*p*-Value
Older age	1.048 (1.016–1.081)	0.003 *	1.038 (1.003–1.074)	0.034 *
Postmenopause	2.650 (1.278–5.497)	0.009 *	1.588 (0.484–5.207)	0.446
Large tumor size	2.512 (1.592–3.964)	<0.001 *	2.272 (1.397–3.696)	0.001 *
Positive lymphovascular invasion	2.792 (1.199–6.497)	0.017 *	1.644 (0.542–4.987)	0.380
Positive lymph node metastasis	2.369 (1.080–5.195)	0.031 *	1.920 (0.426–8.658)	0.396
Stage II vs. Stage I, III	3.435 (1.579–7.473)	0.002 *	0.840 (0.193–3.647)	0.816
Stage III vs. Stage I, II	2.672 (0.872–8.189)	0.085	0.230 (0.021–2.526)	0.229
Positive intratumoral inflammation	4.050 (1.691–9.703)	0.002 *	3.811 (1.435–10.123)	0.007 *
Positive peritumoral inflammation	4.133 (1.562–10.936)	0.004 *	1.764 (0.427–7.291)	0.433

OR = odds ratio; CI = confidence interval; * Indicates statistically significant (*p* < 0.05).

## Data Availability

The datasets used and/or analyzed during the current study are available from the corresponding author upon reasonable request.
